# Expression, purification, crystallization and preliminary X-ray crystallographic studies of a mitochondrial membrane-associated protein Cbs2 from *Saccharomyces cerevisiae*

**DOI:** 10.7717/peerj.10901

**Published:** 2021-02-17

**Authors:** Dan Wu, Guanyu Zhu, Yufei Zhang, Yan Wu, Chunlei Zhang, Jiayi Shi, Xiaofeng Zhu, Xiaohuan Yuan

**Affiliations:** 1 Heilongjiang Key Laboratory of Anti-fibrosis Biotherapy, Mudanjiang Medical University, Mudanjiang, China; 2 Department of Neurosurgery, Beijing Tiantan Hospital, Capital Medical University, Beijing, China; 3 Mudanjiang Medical University, Mudanjiang, China

**Keywords:** Mitochondria, Cytochrome b, Cbs2, Expression and purification, Crystallization

## Abstract

**Background:**

Mitochondria are unique organelles that are found in most eukaryotic cells. The main role of the mitochondria is to produce ATP. The nuclear genome encoded proteins Cbs1 and Cbs2 are located at the mitochondrial inner membrane and are reported to be essential for the translation of mitochondrial cytochrome b mRNA. Genetic studies show that Cbs2 protein recognizes the 5′ untranslated leader sequence of mitochondrial cytochrome b mRNA. However, due to a lack of biochemical and structural information, this biological process remains unclear. To investigate the structural characteristics of how *Saccharomyces cerevisiae* (*S. cerevisiae*) Cbs2 tethers cytochrome b mRNA to the mitochondrial inner membrane, a preliminary X-ray crystallographic study was carried out and is reported here.

**Methods:**

The target gene from *S. cerevisiae* was amplified by polymerase chain reaction. The PCR fragment was digested by the NdeI and XhoI restriction endonucleases and then inserted into expression vector p28. After sequencing, the plasmid was transformed into *Escherichia coli* C43 competent cells. The selenomethionine derivative Cbs2 protein was overexpressed using M9 medium based on a methionine-biosynthesis inhibition method. The protein was first purified to Ni^2+^-nitrilotriacetate affinity chromatography and then further purified by Ion exchange chromatography and Gel-filtration chromatography. The purified Se-Cbs2 protein was concentrated to 10 mg/mL. The crystallization trials were performed using the sitting-drop vapor diffusion method at 16 °C. The complete diffraction data was processed and scaled with the HKL2000 package and programs in the CCP4 package, respectively.

**Results:**

Cbs2 from *S. cerevisiae* was cloned, prokaryotic expressed and purified. The analysis of the size exclusion chromatography showed that the Cbs2 protein peaked at a molecular weight of approximately 90 KDa. The crystal belonged to the space group C2, with unit-cell parameters of *a* = 255.11, *b* = 58.10, *c* = 76.37, and *β* = 95.35°. X-ray diffraction data was collected at a resolution of 2.7 Å. The Matthews coefficient and the solvent content were estimated to be 3.22 Å 3 Da-1 and 61.82%, respectively.

**Conclusions:**

In the present study Cbs2 from *S. cerevisiae* was cloned, expressed, purified, and crystallized for structural studies. The molecular weight determination results indicated that the biological assembly of Cbs2 may be a dimer.The preliminary X-ray crystallographic studies indicated the presence of two Cbs2 molecules in the asymmetric unit. This study will provide an experimental basis for exploring how Cbs2 protein mediates cytochrome b synthesis.

## Introduction

Mitochondria are unique organelles that are present in most eukaryotic cells and are composed of five distinct parts, including the outer mitochondrial membrane, the intermembrane space, the inner mitochondrial membrane, the cristae space, and the matrix (Frey & Mannella, 2000; Griparic & Van der Bliek, 2001). The diameter of a mitochondrion ranges from 0.75–3 µm, varying in size and structure in different species (Wiemerslage & Lee, 2016). The most prominent role of the mitochondria is in the production of the energy currency (ATP) of cells (Pedersen, 1994). In addition, mitochondria are involved in a series of metabolic tasks, including apoptosis (Green, 1998), calcium signaling (Hajnoczky et al., 2006), steroid synthesis (Rossier, 2006), hormonal signaling (Klinge, 2008), and the regulation of cellular metabolism (McBride, Neuspiel & Wasiak, 2006). Mitochondrial proteins are the most important elements involved in these biological processes. Most mitochondrial proteins are synthesized by cytosolic ribosomes and are then imported into the mitochondrion. The mitochondrion has its own genetic material (mtDNA) and ribosomes (mitoribosomes), which are responsible for synthesizing the essential components of the oxidative phosphorylation (OXPHOS) system.

Mitochondrial gene expression in the yeast *S. cerevisiae* is a highly complex process and requires a large number of trans-acting factors. The ribosomes in high eukaryotes scan the 5′ untranslated leader sequence of mRNA and find the translation start site. However, this is hard to do in the yeast mitochondrion, because the mRNAs in this organism usually lack a 5′ cap structure and the 5′ untranslated leader sequence forms stable secondary structures.

The nuclear genome encoded proteins Cbs1 and Cbs2 are essential for the translation of mitochondrial cytochrome b mRNA (Rodel, 1986; Michaelis, Schlapp & Rodel, 1988). Both Cbs1 and Cbs2 are located at the mitochondrial inner membrane (Michaelis, Korte & Rodel, 1991). Cbs1 is an integral membrane protein of 24 kDa, while Cbs2 behaves like a membrane-associated protein, with a molecular weight of 44 kDa. The biochemical information of the Cbs1 protein is incomplete,which may be due to the difficulty in selecting a suitable detergent to stabilize it. The Cbs2 protein is comprised of two domains. The N-terminal portion mediates the formation of homomeric structures, and the C-terminus is vital for mitoribosome association (Krause-Buchholz et al., 2004). Genetic studies show that the Cbs2 protein recognizes the 5′ untranslated leader sequence of mitochondrial cytochrome *b* mRNA (Michaelis & Rodel, 1990).

Mitoribosomes in yeast are not able to recognize mRNA and find the AUG start codon in an unaided manner. Thus, the Cbs1 and Cbs2 proteins are thought to link cytochrome b mRNA to the mitochondrial inner membrane via the 5′ untranslated leader sequence for translation (Krause-Buchholz et al., 2004). However, due to the lack of biochemical and structural information, this biological process remains unclear. To investigate the structural characteristics of how *S. cerevisiae* Cbs2 tethers cytochrome b mRNA to the mitochondrial inner membrane, we performed cloning, expression, purification, crystallization, and preliminary X-ray crystallographic studies using *S. cerevisiae* Cbs2.

## Materials & Methods

### Acquisition of the target Cbs2 gene by PCR amplification

The primers were designed using Primer 5.0 and the Cbs2 full-length sequence (GenBank, NM_001180505.1), and the restriction enzyme sites NdeI and XhoI were added to the upstream and downstream primers. The expected amplified product fragment size was 1,170 bp. The upstream primer, 5′-GCGCATATGTCAAGCTCA-ATACCTAG- 3′ and the downstream primer, 5′-GAGCACTCGAGTCACAGGTAA-TGATAATCTAG- 3′, were synthesized by Shanghai Shenggong Bioengineering Technology Service Co., Ltd. The target Cbs2 gene from *S. cerevisiae* (strain ATCC 204508) was amplified by PCR. The reaction was performed according to the 2xEs Taq MasterMix (CWBIO) instructions. The reaction mixture was 50 µL and consisted of 2x Es Taq MasterMix (25 µL), upstream and downstream primers (10µM, 1 µL each), double-distilled water (22 µL), and template (1 µL). After pre-denaturation at 94 °C for 5 min, 30 cycles of 94 °C for 30 s, 58 °C for 30 s, and 72 °C for 1 min 30 s were performed, followed by a final extension at 72 °C for 7 min. The amplified product was subjected to electrophoresis on a 1.0% agarose gel, and the PCR product was recovered and purified according to the instructions in the Quick Gel Extraction Kit (CWBIO).

### Construction and identification of the p28-Cbs2 plasmid

The PCR product was digested using the NdeI and XhoI restriction endonucleases (Thermo Scientific) at 37 °C for 3 h. The digested products were separated by electrophoresis on a 1.0% agarose gel and were purified using the Quick Gel Extraction Kit (CWBIO). The digested Cbs2 fragment was inserted into the expression vector pET-28a (Novagen). The ligation products were transformed into DH5 *α* competent cells (Novagen) and were cultured overnight on LB-coated plates containing 50 µg/mL Kanamycin. The positive clones were screened by bacterial liquid PCR. The identity of the recombinant plasmid was initially confirmed by PCR and double enzyme digestion. Then, the plasmids were sequenced by Shanghai Shenggong Bioengineering Technology Service Co., Ltd. The obtained cDNA sequences were analyzed by MegAlign and were aligned to the Cbs2 gene sequence in GenBank.

### Efficient Cbs2 protein expression in *Escherichia coli* competent cells

The pET-28a -Cbs2 recombinant plasmid was transformed into *Escherichia coli* C43 (DE3) competent cells (Novagen). The positive colony was inoculated into 10 mL of LB medium containing 50 µg/mL Kanamycin and cultured overnight at 37 °C. Then, the bacterial solution was inoculated into 1L of LB medium at a ratio of 1:200 and was cultured on a shaker at 37 °C. The overexpression of the Cbs2 protein was induced by isopropyl- *β*-D-thiogalactoside (IPTG) at different induction temperatures, IPTG concentrations, and induction times to screen for the best induction conditions.

### Primary purification of the Cbs2 protein by Ni-affinity chromatography

After an overnight culture, the cells were collected by centrifugation at 6000g for 15 min. The harvested cells were suspended in buffer A (20 mM Tris-HCl pH 7.5, 200 mM NaCl, 5% glycerol) and were lysed by sonication on ice. The supernatant was obtained after centrifugation at 12000g for 40 min and was then applied onto a Ni^2+^-nitrilotriacetate affinity resin (Ni-NTA, Qiagen). Impurities were washed away with buffer B (20 mM Tris-HCl pH 7.5, 200 mM NaCl, 5% glycerol, 20 mM imidazole) and buffer C (20 mM Tris-HCl pH 7.5, 200 mM NaCl, 5% glycerol, 40 mM imidazole). The Cbs2 protein was eluted using buffer D (20 mM Tris-HCl pH 7.5, 200 mM NaCl, 5% glycerol, 300mM imidazole). The elution protein was estimated by 12% SDS-PAGE. The identity of the Cbs2 protein was confirmed by Western blot with the mouse anti-His Tag Monoclonal Antibody (Invitrogen, 1:2,000 dilution), followed by HRP-conjugated goat anti-mouse IgG Secondary Antibody (Cell signaling; 1:5000 dilution).

### Further purification of the Cbs2 protein by ion exchange and gel filtration chromatography

The eluted Cbs2 protein was purified by FPLC using a Hitrap Q anion-exchange chromatography column (GE Healthcare) in buffer E (20 mM Tris-HCl, pH 7.5, 5 mM NaCl, 2mM DTT) and buffer F (20 mM Tris-HCl, pH 7.5, 1 M NaCl, 2mM DTT). The peak proteins were collected and estimated by 12% SDS-PAGE.

The collected peak protein was further purified using a Superdex 200 10/300 gel-filtration chromatography column (GE Healthcare) in buffer G (20 mM Tris-HCl, pH 7.5, 100 mM NaCl, 2 mM DTT). The peak Cbs2 protein was estimated by 12% SDS-PAGE. Standard calibration curve establishment and protein molecular weight determination were performed using two Gel Filtration Calibration Kits (HMW and LMW, GE Healthcare) by gel filtration chromatography. The purity of Cbs2 protein after each purification step was estimated by the ratio of the absorption area of the corresponding peak to the total absorption area at 280 nm.

### Selenomethionine derivative Cbs2 (Se-Cbs2) expression and purification

**Figure 1:**
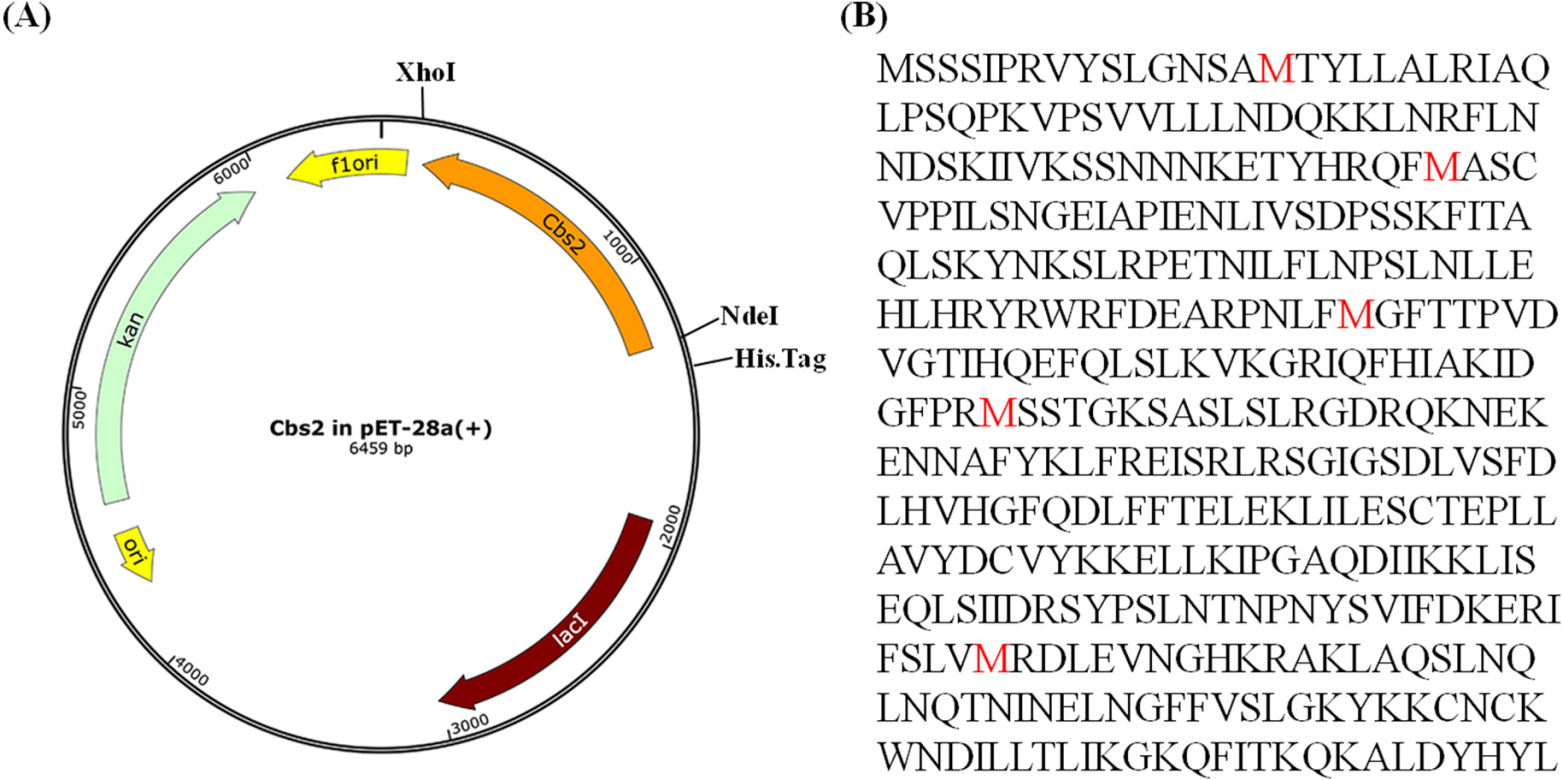
The Cbs2 expression plasmid and the encoded protein. (A) The 6-His tag and the cleavage site are marked with black lines. (B) Sequence of *Saccharomyces cerevisiae* Cbs2 protein. The methionines are labelled in red.

Due to the lack of the known Cbs2 homologous protein structure, the Se-Cbs2 protein was overexpressed using M9 medium based on a methionine-biosynthesis inhibition method (Wu et al., 2017). The methionines are labeled on the *S. cerevisiae* Cbs2 sequence (Fig. 1B). At OD600 of 0.8, the temperature was reduced to 16 °C, and supplemented with 50 µg/mL SeMet (Sigma-Aldrich) and specific amino acids: Ile, Leu and Val at 50 µg/mL , Lys, Phe and Thr at 100 µg/mL. The Se-Cbs2 was purified by the same method as the native Cbs2 described above.

### Preliminary screening and optimized crystallization of the Cbs2 protein

The preliminary screening of the crystallization conditions was carried out at 16 °C using a drop vapor diffusion method in a 24-well plate. Each droplet was prepared by mixing 1.2 µL of the protein solution with 1.2 µL of a reservoir solution and equilibrating against 400 µL of a reservoir solution. A series of crystallization kits were used for the initial crystallization experiments, including Crystal Screen, Crystal Screen 2, ProPlex, Salt RX, Index, PEG/Ion, and PEG/Ion 2 (Hampton). Protein crystallization was observed daily through a microscope. Once the crystals were observed, the relevant crystallization conditions were optimized. The crystallization optimization was mainly focused on the different reagent ratios, protein concentrations, and crystallization temperatures.

### Cbs2 X-ray data collection and processing

For the data collection, all the crystals were soaked in a cryo-protectant solution that consisted of the respective reservoir solution supplemented with 25% (v/v) glycerol, and these were then flash frozen in liquid nitrogen. The X-ray diffraction data was collected at the Shanghai Synchrotron Radiation Facility (SSRF). The complete diffraction data was processed and scaled with the HKL2000 package (Otwinowski & Minor, 1997) and programs in the CCP4 package (Collaborative Computational Project, 1994), respectively.

## Results

### Cloning and prokaryotic expression of the full-length Cbs2 gene

The agarose gel electrophoresis results showed that the size of the PCR product was consistent with the size of the full-length Cbs2 sequence, indicating that the target fragment was successfully amplified (Fig. S1A). The digested Cbs2 fragment was inserted into the pET-28a vector to generate the recombinant Cbs2 with a hexahistidine tag (HHHHHH) at the N-terminus. After transformation into DH5 *α* cells, five colonies were screened by bacterial liquid PCR. Four colonies were positive for amplifying the target fragments (Fig. S1B). Two positive colonies were selected and cultured overnight to extract the plasmids. The plasmid PCR electrophoresis results indicated that the plasmid could be used as a template to successfully amplify the target fragment (Fig. S1C). The results of double-digestion plasmid electrophoresis showed that the digestion product was consistent with the size of the vector and the target fragment (Fig. S1D). These results, combined with the sequencing comparison, indicated that the pET-28a-Cbs2 plasmid was successfully constructed (Fig. 1A). The cloning details are shown in Table S1.

The recombinant Cbs2 plasmid was transformed into *E. coli* C43 (DE3) (Novagen) competent cells. Normal Cbs2 protein was overexpressed using LB medium. Due to the lack of the known C2c1 homologous protein structure, the selenomethionine derivative Cbs2 (Se-Cbs2) protein was overexpressed using M9 medium based on a methionine-biosynthesis inhibition method. The methionines are labeled on the *S. cerevisiae* Cbs2 sequence (Fig. 1B).

**Figure 2:**
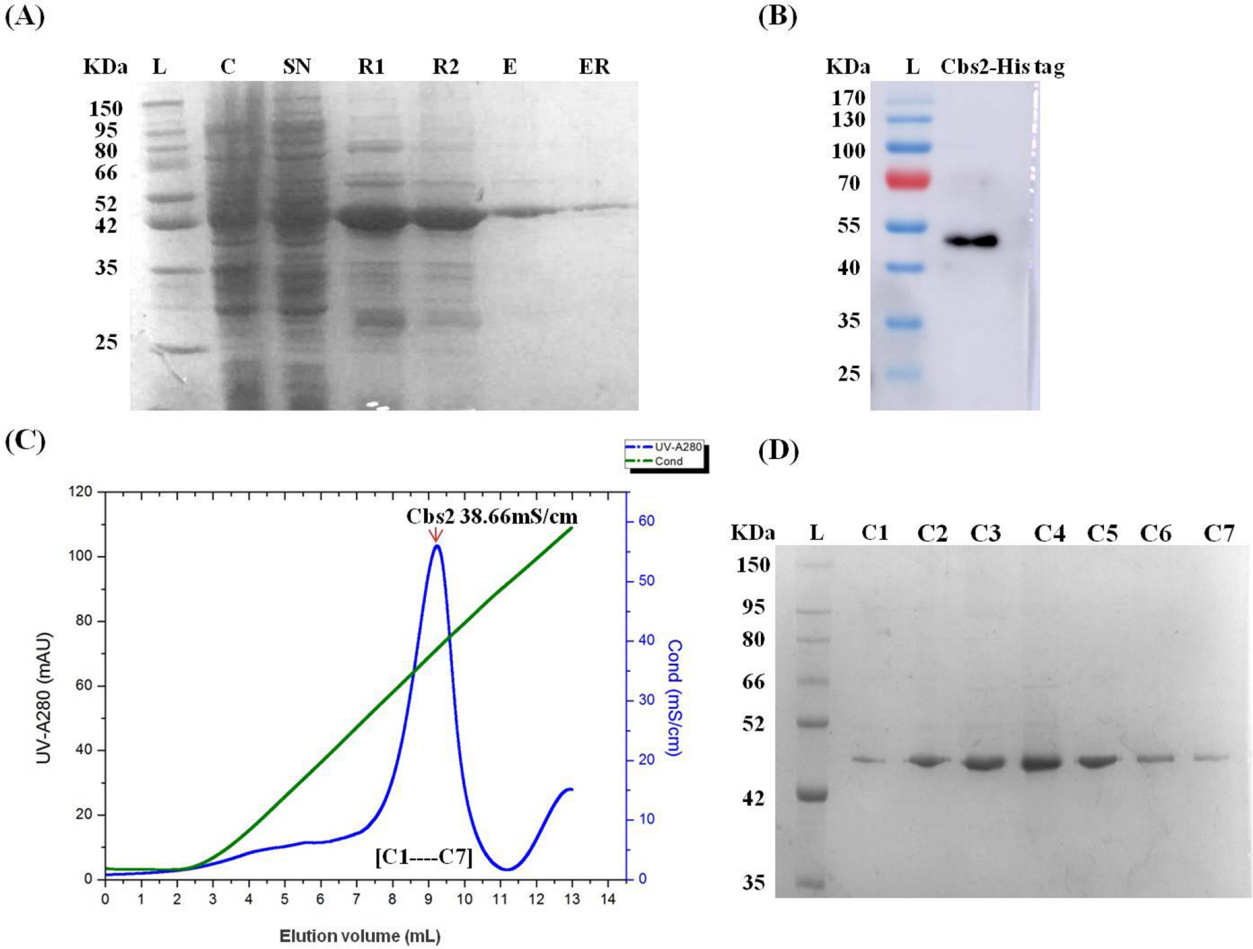
Purification of the Cbs2 protein by Ni NTA affinity chromatography and FPLC Resource Q anion-exchange chromatography (GE Healthcare). (A) SDS-PAGE (12%) analysis of Ni NTA affinity purified Cbs2 protein. L, Ladder; C,Crude; S, Supernatant; R1, Resin washed by 20 mL washing buffer containing 50 mM imidazole; R2, Resin washed by 20 mL washing buffer containing 70 mM imidazole; E, Elution of Cbs2; ER, Resin after elution. (B) Western blot analysis of Ni NTA affinity purified Cbs2 protein. L, Ladder; Cbs2-His tag, Purified Cbs2 protein with His tag. The identity of Cbs2 was confirmed by western blot, and as expected, the Cbs2 protein was recognized by mouse anti-His6 antibody IgG, followed by HRP-conjugated goat anti-mouse IgG Ab. (C) Hitrap Q anion-exchange chromatography profile of Cbs2 protein. Peak Cbs2 38.66mS/cm represents that Cbs2 protein was eluted when the conductance is 38.66 mS/cm. The green line, the gradient of the concentration of NaCl increased from 0.05M to 1 M. (D) SDS-PAGE (12%) analysis of Cbs2 protein purified by Hitrap Q. L, Ladder; C1–C7, fractions C1–C7 of Peak Cbs2.

After culturing at 37 °C until the OD600 was 0.8, 0.5 mM IPTG was added for an overnight induction. Although the Cbs2 protein was expressed, there were many impurities and less soluble expression. The impurities were unable to be washed away even after using a washing buffer that contained up to 70 mM imidazole (Figs. S2A and S2B). Thus, the purity of the protein after purification by the ion exchange column was still not high, and the protein behavior was not good (Figs. S2C and S2D). The reason for this might be because the recombinant protein was expressed too fast in *E. coli* and was not able to fold normally, resulting in aggregation and inclusion bodies. Therefore, we improved the induction conditions and slowed down the synthesis rate of the protein by adding 0.3 mM IPTG for 20 h after cooling it down to 16 °C. The results showed that long-term induction at low temperature facilitated the correct folding of the protein, reduced the production of inclusion bodies and impurities, and increased the expression of soluble proteins. The SDS-PAGE showed that the molecular weight of the Cbs2 protein was about 44 KDa, which was consistent with the expectations (Fig. 2A).

### Multi-step purification and molecular weight determination of the Cbs2 protein

After optimizing the induced expression conditions, the protein was initially purified by Ni-affinity chromatography. The electrophoresis results showed that the protein was expressed in the whole bacteria and the supernatant. After loading, the nickel column was washed using buffers B and C to clean most of the impurities. The elution buffer D was successfully used to obtain the initial purification purpose protein (Fig. 2A). The identity of the eluted protein was confirmed by a Western blot, and as expected, the Cbs2 protein was recognized by the mouse anti-His Tag monoclonal antibody (Fig. 2B). The eluted Cbs2 protein was purified by anion-exchange chromatography. The NaCl concentration gradient increased from 5 mM to 1 M. Peak Cbs2 38.66 mS/cm represents the Cbs2 protein that was eluted when the conductance was 38.66 mS/cm (Figs. 2C and 2D).

**Figure 3:**
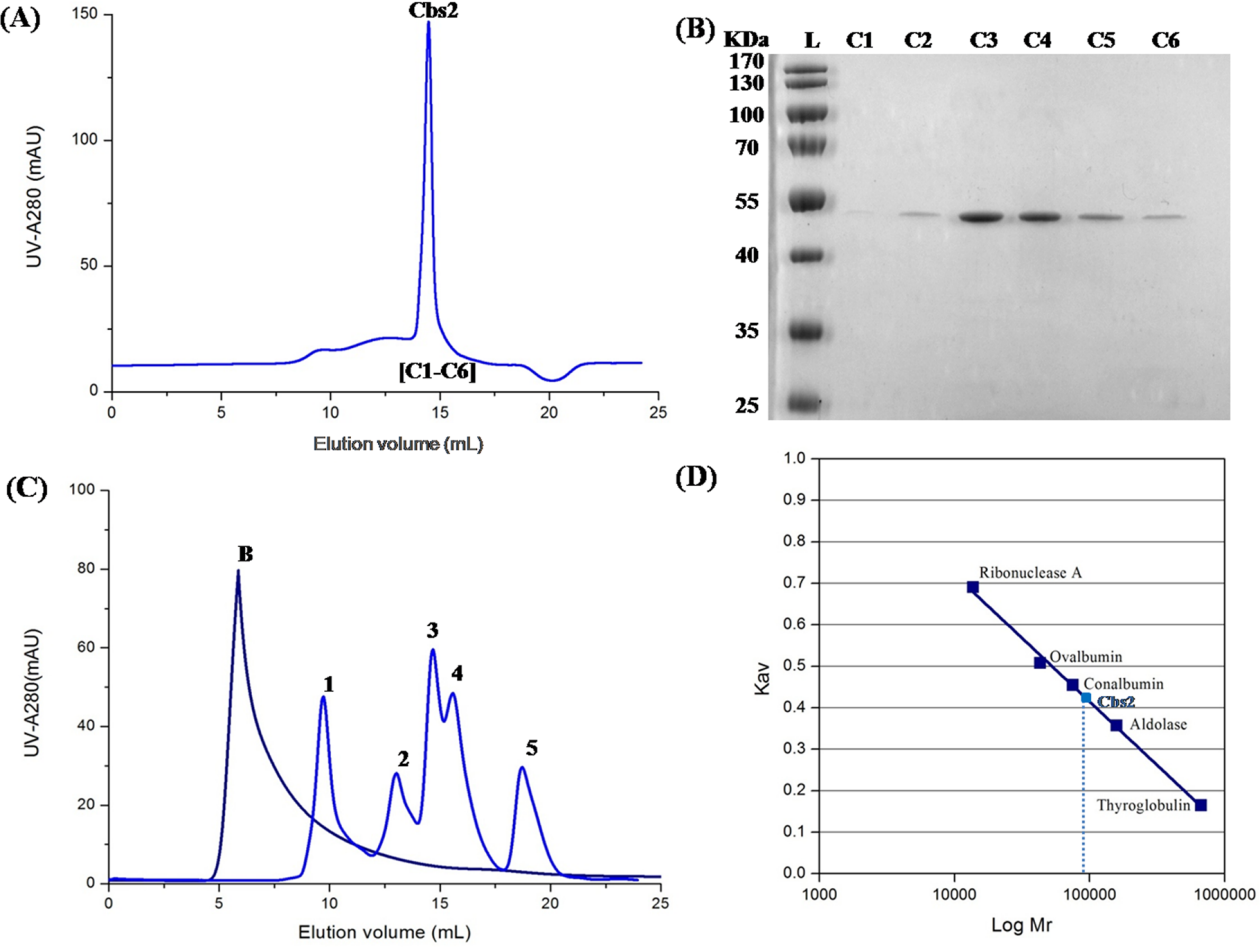
Further purification of the Cbs2 protein by Superdex 200 10/300 gel-filtration chromatography colum (GE Healthcare). (A) Size-exclusion chromatography profile of Cbs2 protein . Peak Cbs2 represents further purified Cbs2 protein. (B) SDS-PAGE (12%) analysis of Cbs2 protein purified by Superdex 200 10/300. L, Ladder; C1–C6, fractions C1–C6 of Peak Cbs2. (C) Standard chromatography profile determined by Gel Filtration Calibration Kit The protein standard peaks are: 1, thyroglobulin (669 kDa); 2, aldolase (158 kDa); 3, conalbumin (75 kDa); 4, ovalbumin (44 kDa) and 5, Ribonuclease A(13.7 kDa). B, Blue dextran (2,000 kDa) was used to determine the void volume (Vo) of the column. (D) The calibration curve is shown on the right side. Partition coefficient (Kav) was calculated from the formula, Kav = (VE−V0)/(VT−V0), where VE is the retention volume of each sample, VT is the total column volume, and V0 is the void volume, respectively. Kav was plotted against the molecular weight of proteins.

The collected peak protein was further purified using a gel filtration chromatography column. The peak Cbs2 protein was concentrated to 10 mg/mL and estimated by SDS-PAGE (Figs. 3A and 3B). Standard calibration curve establishment and protein molecular weight determination were performed by gel filtration chromatography (Figs. 3C and 3D). The protein standard peaks were as follows: 1, thyroglobulin (669 kDa); 2, aldolase (158 kDa); 3, conalbumin (75 kDa); 4, ovalbumin (44 kDa), and 5, Ribonuclease A (13.7 kDa). Blue dextran (2000 kDa) was used to determine the void volume (Vo) of the column. The calibration curve is shown on the right side. The partition coefficient (Kav) was calculated using the formula: Kav = (VE–V0)/(VT–V0), where VE is the retention volume of each sample, VT is the total column volume, and V0 is the void volume. The Kav was plotted against the molecular weights of the proteins. The analysis of the size exclusion chromatography showed that the Cbs2 protein peaked at a molecular weight of approximately 90 KDa. The results indicated that the biological assembly of Cbs2 may be a dimer. The protein yield (mg/L of cell culture) and purity (%) of each purification step are shown in Table S2.

### Se-Cbs2 optimized crystallization

Crystals were observed under several conditions over 8 days. The primary screened crystals were smaller, and this crystallization conditions needed to be optimized. The optimization work was initially focused on expanding around the two reagents (dipotassium hydrogen phosphate and polyethylene glycol 3350). The optimization process included different protein concentrations (10 mg/mL, 8 mg/mL, 6 mg/mL, and 4 mg/mL) and crystallization temperatures (20 °C, 16 °C, and 4 °C). After the optimization, single crystals suitable for the X-ray diffraction measurement were grown in droplets containing 0.2 M dipotassium hydrogen phosphate and 18% polyethylene glycol 3350 at 16 °C. The crystallization details are shown in Table S3.

### Se-Cbs2 preliminary X-ray crystallographic studies

**Figure 4:**
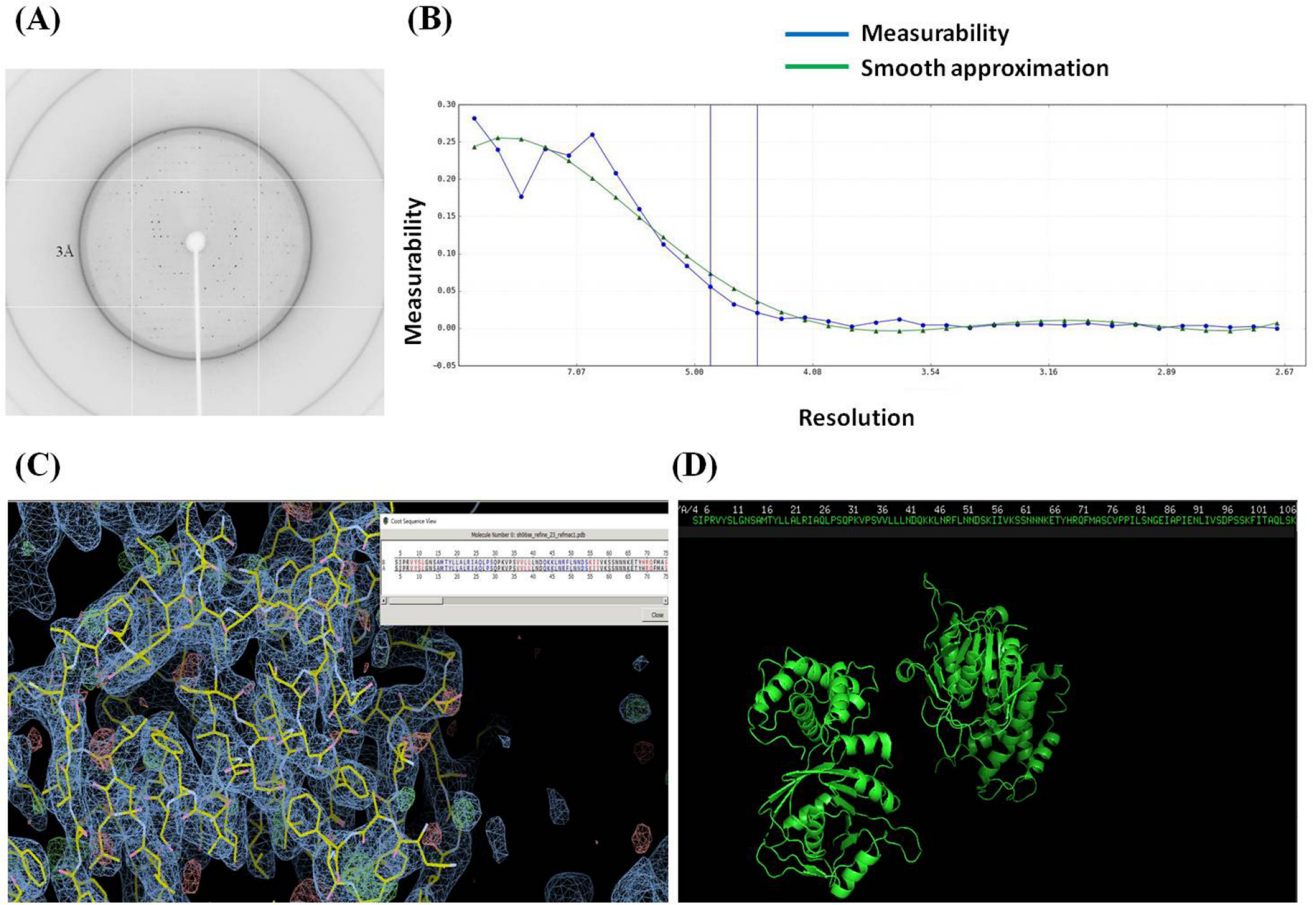
Crystallization and diffraction Data Collection of the recombinant Cbs2. (A) A diffraction image of Cbs2 protein collected on beamline 17U1 at the Shanghai Synchrotron Radiation Facility (SSRF) at 100 K. (B) Measurability of anomalous signal. (C) Electron density map of Cbs2. (D) Cartoon representation of Cbs2.

The X-ray diffraction data was collected on a beamline 17U1 at the Shanghai Synchrotron Radiation Facility (SSRF) at 100 K. All the frames were collected using a 1° oscillation angle at a wavelength of 0.9792 Å. A total of 360 diffraction images were recorded from a single crystal (Fig. 4A). The crystal-to-detector distance was set at 350 mm. The measurability of anomalous signal is shown in Fig. 4B. The electron density map and cartoon representation of Cbs2 are shown in Figs. 4C and 4D. The statistics for the diffraction data are summarized in Table 1. The crystal belongs to the space group C2, with unit-cell parameters of *a* = 255.11, *b* = 58.10, *c* = 76.37, and *β* = 95.35°. The Matthews coefficient was calculated by the CCP4 suite, which suggested the presence of two Cbs2 molecules in an asymmetric unit. The X-ray diffraction data was processed at a resolution of 2.7 Å. Structure determination is currently in progress.

**Table 1:** Data collection and refinement statistics of Cbs2.

Data collection statistics	Se-Cbs2
Diffraction source	BL17U1, SSRF
Wavelength (Å)	0.9792
Temperature (K)	100
Detector	Jupiter CCD
Crystal-detector distance (mm)	350 mm
Rotation range per image (°)	1
Total rotation range (°)	360
Exposure time per image (s)	1
Space group	C2
*a*, *b*, *c* (Å)	255.11, 58.10. 76.37
*α*, *β*, *γ* (°)	90.00, 95.35, 90.00
Mosaicity (°)	0.42
Resolution range (Å)	50.00–2.70 (2.75–2.70)
Total No. of reflections	232330
No. of unique reflections	31035
Completeness (%)	99.8 (99.9)
^B^*R*_merge_ (%)	10.6 (87.4)
*R*_p.i.m_ (%)	4.2 (34.3)
〈*I*∕*σ*(*I*)〉	17.8 (2.6)
Overall *B* factor from Wilson plot (Å^2^)	64.1

**Notes.**

a Values in parentheses are for the highest resolution shell.

b *R*_merge_ = ∑_*hkl*_∑_*i*_|*I*_*i*_(*hkl*) − 〈*I*(*hkl*)〉|∕∑_*hkl*_∑_*i*_*I*_*i*_〈*hkl*〉, where ∑_*hkl*_ is the sum over all reflections and ∑_*i*_ is the sum over all equivalent and symmetry-related reflections.

## Discussion

The respiratory chain of eukaryotic cells is located on the inner mitochondrial membrane and consists of five transmembrane protein complexes (I-V) (Saraste, 1999). Mitochondrial respiratory chain complex I (NADH oxidase) and mitochondrial respiratory chain complex II (succinate dehydrogenase) are the main elements by which electrons enter the mitochondrial electron transport chain (ETC). Complex I catalyzes the oxidation of NADH, and complex II catalyzes the oxidation of succinic acid to fumaric acid. Subsequently, coenzyme Q forms coenzyme (QH2), which eventually leads to a decrease in the terminal electron acceptor O_2_. Mitochondrial respiratory chain complex III (cytochrome c reductase) is a key component of mitochondrial oxidative phosphorylation, is the gatekeeper of the mitochondrial respiratory chain, and is the main source of reactive oxygen species. Mitochondrial respiratory chain complex IV (cytochrome c oxidase) is the terminal electron acceptor of the mitochondrial electron transport chain. Complex IV converts O_2_ into water through the oxidation of cytochrome c. This process is related to the synthesis of ATP in the mitochondrial cell membrane (Rich & Marechal, 2010). Mitochondrial respiratory chain complex V and the above four complexes complete oxidative phosphorylation to generate ATP (Griparic & Van der Bliek, 2001).

Most of the proteins needed by organisms are synthesized under the control of genes in the nucleus, and protein translation is mainly carried out in the ribosome in the cytoplasm or endoplasmic reticulum. In addition, mitochondria also contain RNA, DNA polymerase, a complete set of equipment for DNA replication, transcription, and protein translation, such as RNA polymerase and ribosomes, which encode a small amount of protein for the body. However, protein synthesis in mitochondria requires the expression of activators to assist in its completion. Most of these expression activators are encoded by genes in the nucleus, which are then transported into the mitochondria. For example, the normal synthesis of cytochrome b, cytochrome c, and other proteins encoded in the mitochondria requires the assistance of the expression of activating factors (Hartl et al., 1989). When these expression activators are abnormal or absent, even if the mRNA level of the target they regulate is normal, the protein cannot be expressed normally.

Respiratory chain complex III (cytochrome c reductase complex) is a multi-subunit membrane protein complex that exists as a dimer under physiological conditions. The core of each complex III contains a cytochrome b, a cytochrome c1, and an iron-sulfur protein. These three core protein subunits are responsible for the oxidation of hydroquinone, the product of complex I and complex II. In this process, protons are released into the mitochondrial membrane space, and the electron is then transferred to the cytochrome c in the membrane space to complete the electron transfer and the reduction of cytochrome c. Cytochrome b is an important component of respiratory chain complex III (Ndi et al., 2018). Mitochondrial respiratory chain complex III in *Saccharomyces cerevisiae* consists of nine subunits, eight of which are encoded by nuclear genes, and cytochrome b is encoded by mitochondrial COB genes. The transcription of the COB gene starts from the promoter upstream of the glutamine-tRNA gene, and the resulting transcript undergoes multiple processing steps to produce mature COB mRNA and tRNA. The translation of COB pre-mRNA splicing intermediates is a necessary prerequisite for the formation of mature COB mRNA and the synthesis of cytochrome b. According to reports, the nuclear genome-encoded proteins Cbs1 and Cbs2, located in the inner membrane of mitochondria, are directly involved in the processing and maturation of COB pre-mRNA and are essential for the translation of mitochondrial cytochrome b mRNA. The translation activation effect of CBS1 and CBS2 is mediated by the 5′-untranslated leader region (5′-UTL) of COB mRNA (Rodel, 1986; Michaelis, Korte & Rodel, 1991). Mitochondrial ribosomes in yeast may not be able to independently recognize mRNA and find the AUG start codon. Therefore, researchers believe that the CBS1 and CBS2 proteins recognize and bind to the 5′-UTL of the COB mRNA, thereby guiding COB mRNA into the ribosomes located in the inner mitochondrial membrane for translation (Krause-Buchholz et al., 2004).

The architecture of mRNAs in yeast is different from that in high eukaryotes. Yeast mRNA lacks a 5′ cap structure, and furthermore, the 5′ untranslated leader sequence can form stable secondary structures. These features make it difficult to find the initiation codon in yeast mitoribosomes without assistance. Many mitochondrial proteins are identified as trans-acting factors in yeast, which are involved in mitochondrial gene expression. The Cbs1 and Cbs2 proteins located in the inner mitochondrial membrane are essential for the translation of cytochrome b mRNA.

Studies have shown that the Cbs1 gene encodes a protein of 233 amino acids with a net positive charge. Cbs1 is a membrane protein whose amino terminal sequence has the characteristics of a mitochondrial targeting sequence, that is, several positively charged amino acid residues, non-acidic residues, etc.; Cbs2 gene encodes a protein of 389 amino acids, which is positively charged The main amino acids. Cbs2 is a membrane-associated protein that is loosely related to the membrane through electrostatic force . The truncation and mutation of the C-terminal region of Cbs2 will make the protein lose its function, and the amino-terminal amino acid of Cbs2 is important for mitochondrial targeting. Interestingly, Cbs2 is annotated to the function of ketopentoate reductase in the gene function prediction of the Go database (Gaudet et al., 2011). However, there is no related function report at present, and its function needs to be revealed through structural analysis and further biochemical experiments. Researchers have proved that both Cbs1 and Cbs2 interact with mitochondrial ribosomes through experiments such as density gradient centrifugation and affinity chromatography. The BN-PAGE experiment found that the two proteins have almost the same separation profile with overlapping signals, indicating that Cbs1 and Cbs2 may be related to the same high molecular weight complex (mitochondrial ribosomal or ribosomal subunit) (Krause-Buchholz et al., 2005).

Cytochrome b is an important component of respiratory chain complex III. Whether cytochrome b is normally expressed is related to the function of respiratory chain complex III. Studies have shown that: Cbs1 and Cbs2 proteins can regulate the synthesis of cytochrome b. Researchers have conducted a series of studies on the properties and functions of the two proteins, but there is still a lack of strong biochemical and structural information, and the mechanism of this biological process is still unclear. And how do Cbs1 and Cbs2 proteins anchor on the inner mitochondrial membrane? How do Cbs1 and Cbs2 recognize the 5′ -UTL of COB mRNA? How to guide the translation of COB mRNA? Is there a synergy between the two proteins?

To better understand these functions, we purified and crystallized the two proteins. Here, Cbs2 was purified and crystallized for preliminary X-ray crystallography studies, while studies regarding Cbs1 are still in progress. In this study, full-length recombinant Se-Cbs2 was expressed and crystallized to solve the phase problem. A sequence search was performed in the Protein Data Bank (PDB). No homologs of *S. cerevisiae* Cbs2 were found, indicating that the Cbs2 protein may possess novel structural features. The crystal belongs to the space group C2, with unit-cell parameters of *a* = 255.11, *b* = 58.10, *c* = 76.37, and *β* = 95.35°. The Matthews coefficient was calculated by the CCP4 suite, which suggests the presence of two Cbs2 molecules in an asymmetric unit. The X-ray diffraction data was processed at a resolution of 2.7 Å. We are currently assembling the Cbs2-target RNA complex and analyzing the structure of the complex. These studies will help to reveal the biological process of Cbs2 in mediating the synthesis of cytochrome b through the 5′ untranslated leader sequence.

## Conclusions

In the present study Cbs2 from *Saccharomyces cerevisiae* was cloned, expressed in a prokaryote, purified, and crystallized for structural studies. The molecular weight determination and preliminary X-ray crystallographic studies indicated the presence of two Cbs2 molecules in the asymmetric unit. This study will provide an experimental basis for exploring how Cbs2 protein mediates cytochrome b synthesis.

##  Supplemental Information

10.7717/peerj.10901/supp-1 Figure S1 Construction of p28-Cbs2 recombinant plasmid (A) The agarose gel electrophoresis analysis of Cbs2 gene PCR amplification. M, DL2000 DNA Marker; 1, full-length Cbs2 PCR product. (B) The agarose gel electrophoresis analysis of bacterial liquid PCR. M, DL2000 DNA Marker; 1-5, bacterial liquid PCR products of five colonies. (C) The agarose gel electrophoresis analysis of plasmid PCR. M, DL2000 DNA Marker; 1-2, plasmid PCR products of two positive colonies. (D) The agarose gel electrophoresis analysis of plasmid double digestion. M, DS5000 DNA Marker; 1-2, plasmid double digestion products of two positive colonies.

10.7717/peerj.10901/supp-2 Figure S2 Purification of Cbs2 protein before optimized induction (A) SDS-PAGE (12%) Analysis of Ni-NTA affinity purified Cbs2 protein after overnight induction at 37 °C. L, Ladder; C,Crude; S, Supernatant; R1, Resin washed by 10 mL washing buffer containing 15 mM imidazole; R2, Resin washed by 10 mL washing buffer containing 30 mM imidazole; R3, Resin washed by 10mL washing buffer containing 40 mM imidazole; R4, Resin washed by 10 mL washing buffer containing 50mM imidazole; R5, Resin washed by 10 mL washing buffer containing 60mM imidazole; R6, Resin washed by 10 mL washing buffer containing 70 mM imidazole. (B) SDS-PAGE (12%) analysis of Cbs2 protein purified before optimized induction. E, Elution of Cbs2; Q, Eluted protein diluted with buffer B for Hitrap Q . (C) Hitrap Q anion-exchange chromatography profile of Cbs2 protein before optimized induction. The green line, the gradient of the concentration of NaCl increased from 0.05 M to 1 M. (D) SDS-PAGE (12%) analysis of Cbs2 protein purified by Hitrap Q before optimized induction. L, Ladder; C1-C9, fractions C1-C7 of Peak Cbs2.

10.7717/peerj.10901/supp-3 Figure S3 PCR detection of the target Cbs2 gene The agarose gel electrophoresis results showed that the size of the PCR product was consistent with the size of the full-length Cbs2 sequence, indicating that the target fragment was successfully amplified, as shown in Fig. S1A.

10.7717/peerj.10901/supp-4 Figure S4 Bacterial liquid PCR of the Cbs2 colonies After transformation into DH5 *α* cells, five colonies were screened by bacterial liquid PCR. The agarose gel electrophoresis results showed that four colonies were positive for amplifying the target fragments, as shown in Fig. S1B.

10.7717/peerj.10901/supp-5 Figure S5 Plasmid PCR detection of the recombinant Cbs2 plasmid Two positive colonies were selected and cultured overnight to extract the plasmids. The plasmid PCR electrophoresis results indicated that the plasmid could be used as a template to successfully amplify the target fragment, as shown in Fig. S1C.

10.7717/peerj.10901/supp-6 Figure S6 Double-digestion plasmid detection of the recombinant Cbs2 plasmid The double-digestion plasmid electrophoresis results showed that the digestion product was consistent with the size of the vector and the target fragment, as shown in Fig. S1D.

10.7717/peerj.10901/supp-7 Figure S7 SDS-PAGE analysis of the eluted Cbs2 protein The SDS-PAGE results showed that the molecular weight of the Cbs2 protein was about 44KDa, which was consistent with the expectations, as shown in Fig. 2A.

10.7717/peerj.10901/supp-8 Figure S8 Western blot of the eluted Cbs2 protein The identity of the eluted protein was confirmed by a Western blot, and as expected, the Cbs2 protein was recognized by the mouse anti-His Tag monoclonal antibody, as shown in Fig. 2B.

10.7717/peerj.10901/supp-9 Figure S9 SDS-PAGE analysis of anion-exchange purified Cbs2 protein The eluted Cbs2 protein was purified by anion-exchange chromatography. Peak Cbs2 38.66 mS/cm represents the Cbs2 protein that was eluted when the conductance was 38.66 mS/cm, The peak Cbs2 protein was estimated by SDS-PAGE, The SDS-PAGE results showed that the Cbs2 protein was further purified, as shown in Fig. 2D.

10.7717/peerj.10901/supp-10 Figure S10 SDS-PAGE analysis of gel filtration purified Cbs2 protein The collected peak protein was further purified using a gel filtration chromatography column. The peak Cbs2 protein was estimated by SDS-PAGE, The SDS-PAGE results showed that the protein was pure and concentrated, as shown in Fig. 3B.

10.7717/peerj.10901/supp-11 Figure S11 SDS-PAGE analysis of Ni-NTA affinity purified Cbs2 protein after overnight induction at 37 °C The SDS-PAGE results showed that some impurities were unable to be washed away even after using a washing buffer that contained up to 70 mM imidazole, as shown in Fig. S2A.

10.7717/peerj.10901/supp-12 Figure S12 SDS-PAGE analysis of the eluted Cbs2 protein before optimized induction The SDS-PAGE results showed that there were still some impurities in the eluted Cbs2 protein, as shown in Fig. S2B.

10.7717/peerj.10901/supp-13 Figure S13 SDS-PAGE analysis of Cbs2 protein purified by Hitrap Q before optimized induction The SDS-PAGE results showed that the purity of the protein after ion exchange was still not high, and the protein behavior was not good, as shown in Fig. S2D.

10.7717/peerj.10901/supp-14 Table S1 Information of Cbs2 cloning The cloning details were shown, including primers, vector, Cbs2 gene sequences and competent cells.

10.7717/peerj.10901/supp-15 Table S2 Information of Cbs2 purification The protein yield (mg/L of cell culture) and purity (%) of each purification step.

10.7717/peerj.10901/supp-16 Table S3 Information of Cbs2 Crystallization The crystallization details are shown, including method, plate type, temperature, protein concentration, crystallization kits, composition of reservoir solution, volume and ratio of drop and volume of reservoir.

## Additional Information and Declarations

### Data Availability

The following information was supplied regarding data availability: Raw data are available in the Supplemental Files.
