# Supplementary figures and images for: Expression, purification, crystallization and preliminary X-ray crystallographic studies of a mitochondrial membrane-associated protein Cbs2 from Saccharomyces cerevisiae
Source: PeerJ. 2021 Feb 17;9:e10901. doi: 10.7717/peerj.10901 (PMC7896505; doi:10.7717/peerj.10901)

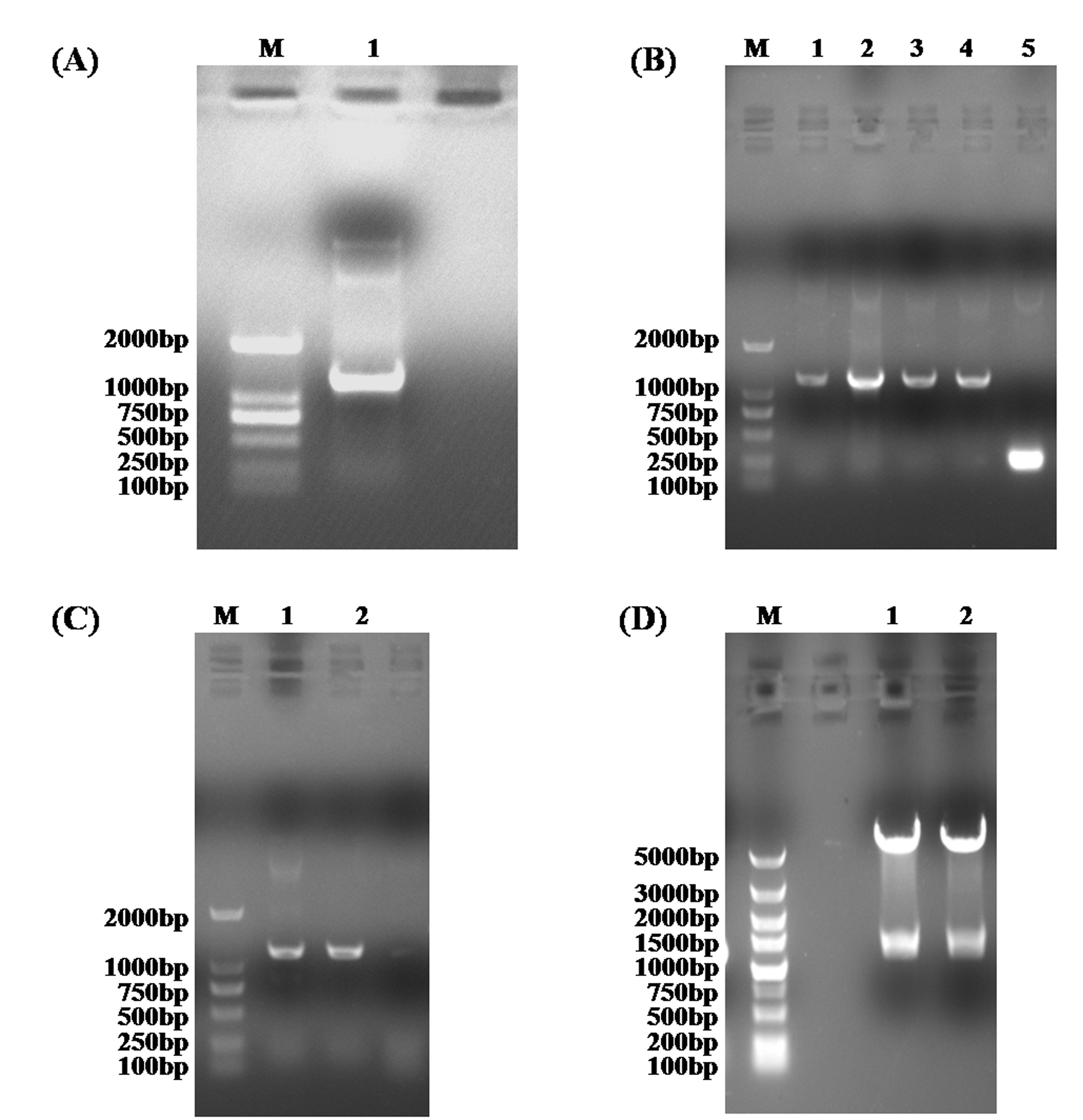

Supplement: Figure S1 — (A) The agarose gel electrophoresis analysis of Cbs2 gene PCR amplification. M, DL2000 DNA Marker; 1, full-length Cbs2 PCR product. (B) The agarose gel electrophoresis analysis of bacterial liquid PCR. M, DL2000 DNA Marker; 1-5, bacterial liquid PCR products of five colonies. (C) The agarose gel electrophoresis analysis of plasmid PCR. M, DL2000 DNA Marker; 1-2, plasmid PCR products of two positive colonies. (D) The agarose gel electrophoresis analysis of plasmid double digestion. M, DS5000 DNA Marker; 1-2, plasmid double digestion products of two positive colonies. [file peerj-09-10901-s001.png]

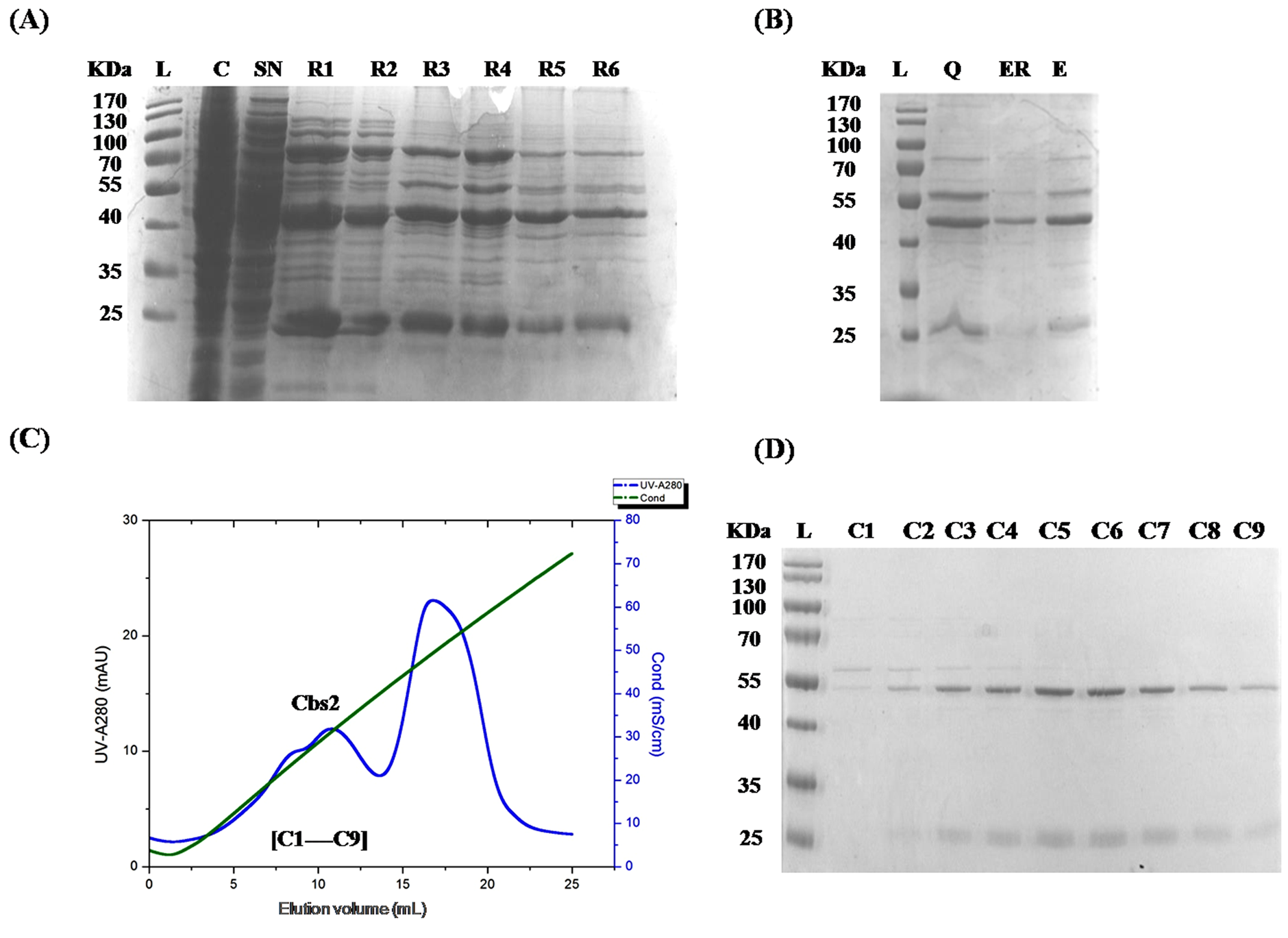

Supplement: Figure S2 — (A) SDS-PAGE (12%) Analysis of Ni-NTA affinity purified Cbs2 protein after overnight induction at 37 °C. L, Ladder; C,Crude; S, Supernatant; R1, Resin washed by 10 mL washing buffer containing 15 mM imidazole; R2, Resin washed by 10 mL washing buffer containing 30 mM imidazole; R3, Resin washed by 10mL washing buffer containing 40 mM imidazole; R4, Resin washed by 10 mL washing buffer containing 50mM imidazole; R5, Resin washed by 10 mL washing buffer containing 60mM imidazole; R6, Resin washed by 10 mL washing buffer containing 70 mM imidazole. (B) SDS-PAGE (12%) analysis of Cbs2 protein purified before optimized induction. E, Elution of Cbs2; Q, Eluted protein diluted with buffer B for Hitrap Q . (C) Hitrap Q anion-exchange chromatography profile of Cbs2 protein before optimized induction. The green line, the gradient of the concentration of NaCl increased from 0.05 M to 1 M. (D) SDS-PAGE (12%) analysis of Cbs2 protein purified by Hitrap Q before optimized induction. L, Ladder; C1-C9, fractions C1-C7 of Peak Cbs2. [file peerj-09-10901-s002.png]

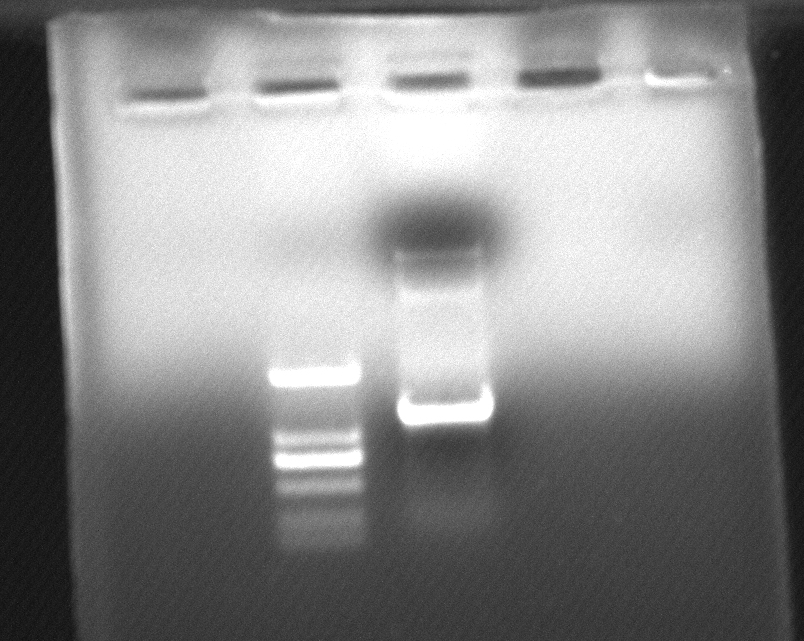

Supplement: Figure S3 — The agarose gel electrophoresis results showed that the size of the PCR product was consistent with the size of the full-length Cbs2 sequence, indicating that the target fragment was successfully amplified, as shown in Fig. S1A. [file peerj-09-10901-s003.jpg]

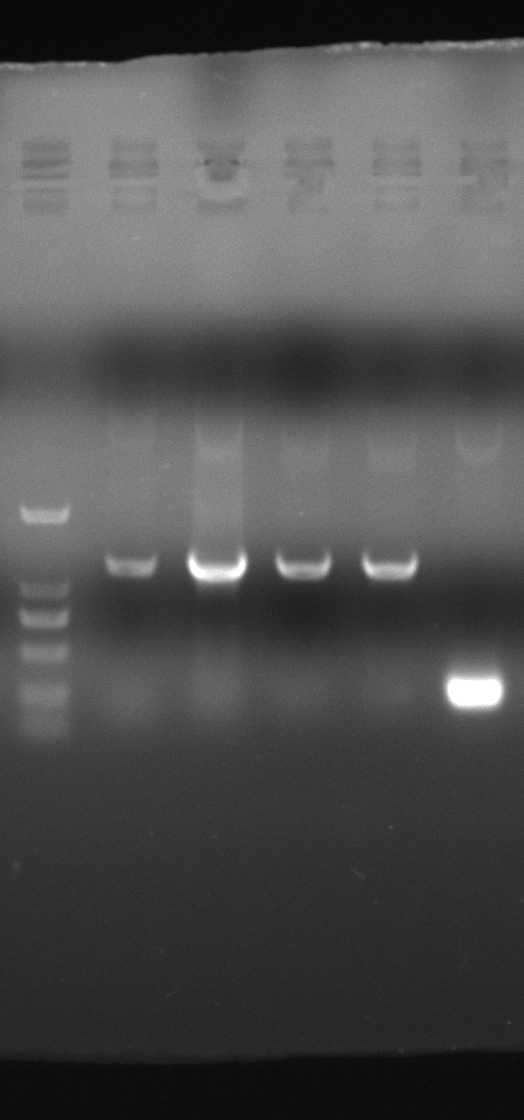

Supplement: Figure S4 — After transformation into DH5 α cells, five colonies were screened by bacterial liquid PCR. The agarose gel electrophoresis results showed that four colonies were positive for amplifying the target fragments, as shown in Fig. S1B. [file peerj-09-10901-s004.jpg]

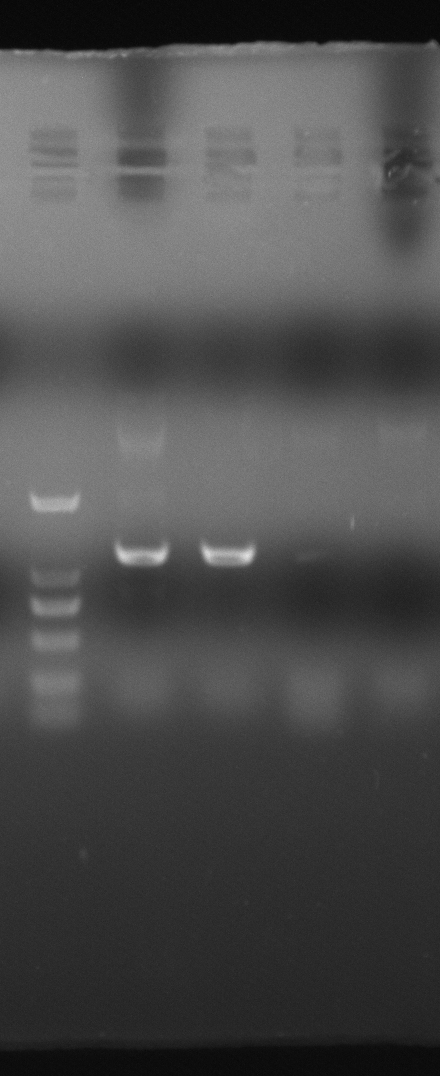

Supplement: Figure S5 — Two positive colonies were selected and cultured overnight to extract the plasmids. The plasmid PCR electrophoresis results indicated that the plasmid could be used as a template to successfully amplify the target fragment, as shown in Fig. S1C. [file peerj-09-10901-s005.jpg]

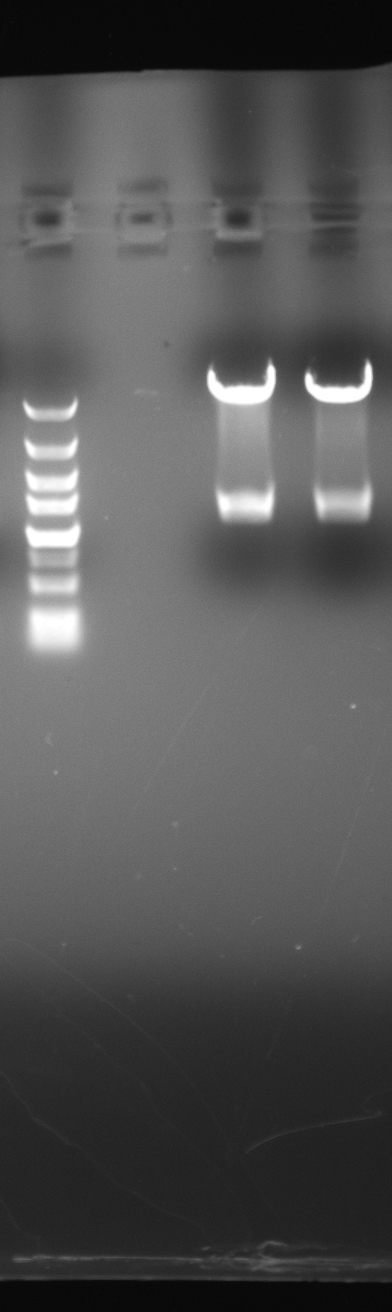

Supplement: Figure S6 — The double-digestion plasmid electrophoresis results showed that the digestion product was consistent with the size of the vector and the target fragment, as shown in Fig. S1D. [file peerj-09-10901-s006.jpg]

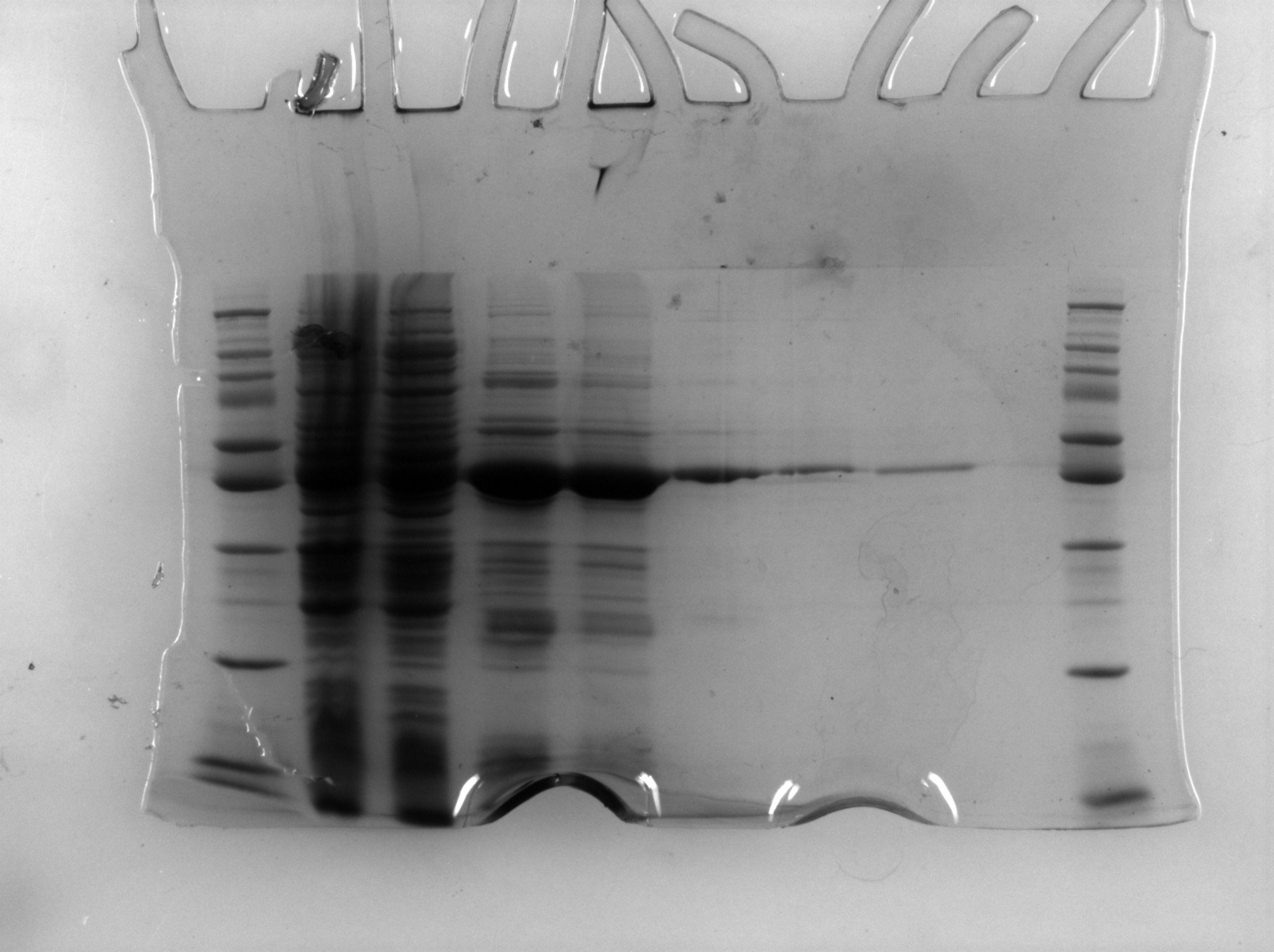

Supplement: Figure S7 — The SDS-PAGE results showed that the molecular weight of the Cbs2 protein was about 44KDa, which was consistent with the expectations, as shown in Fig. 2A. [file peerj-09-10901-s007.jpg]

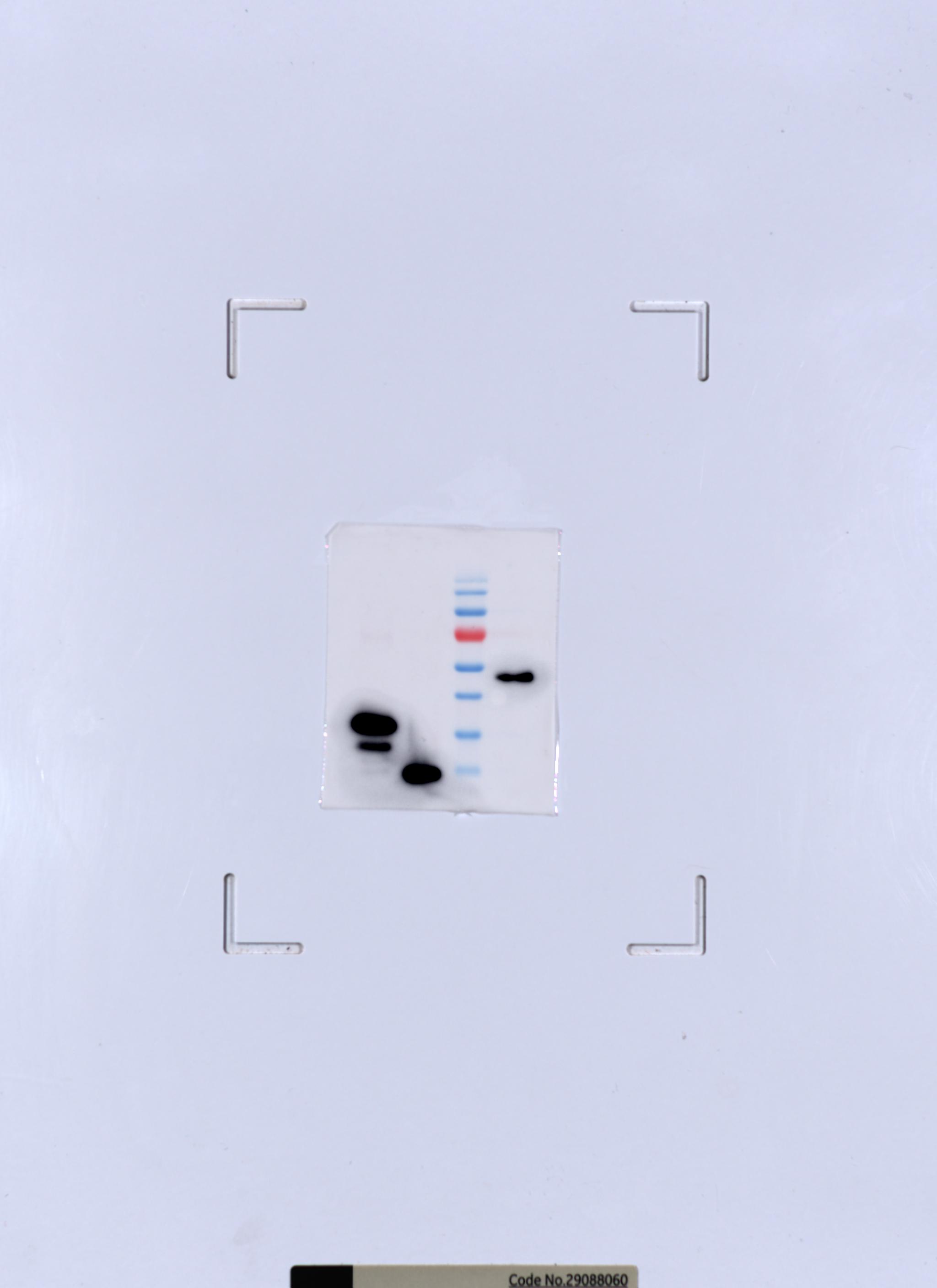

Supplement: Figure S8 — The identity of the eluted protein was confirmed by a Western blot, and as expected, the Cbs2 protein was recognized by the mouse anti-His Tag monoclonal antibody, as shown in Fig. 2B. [file peerj-09-10901-s008.jpg]

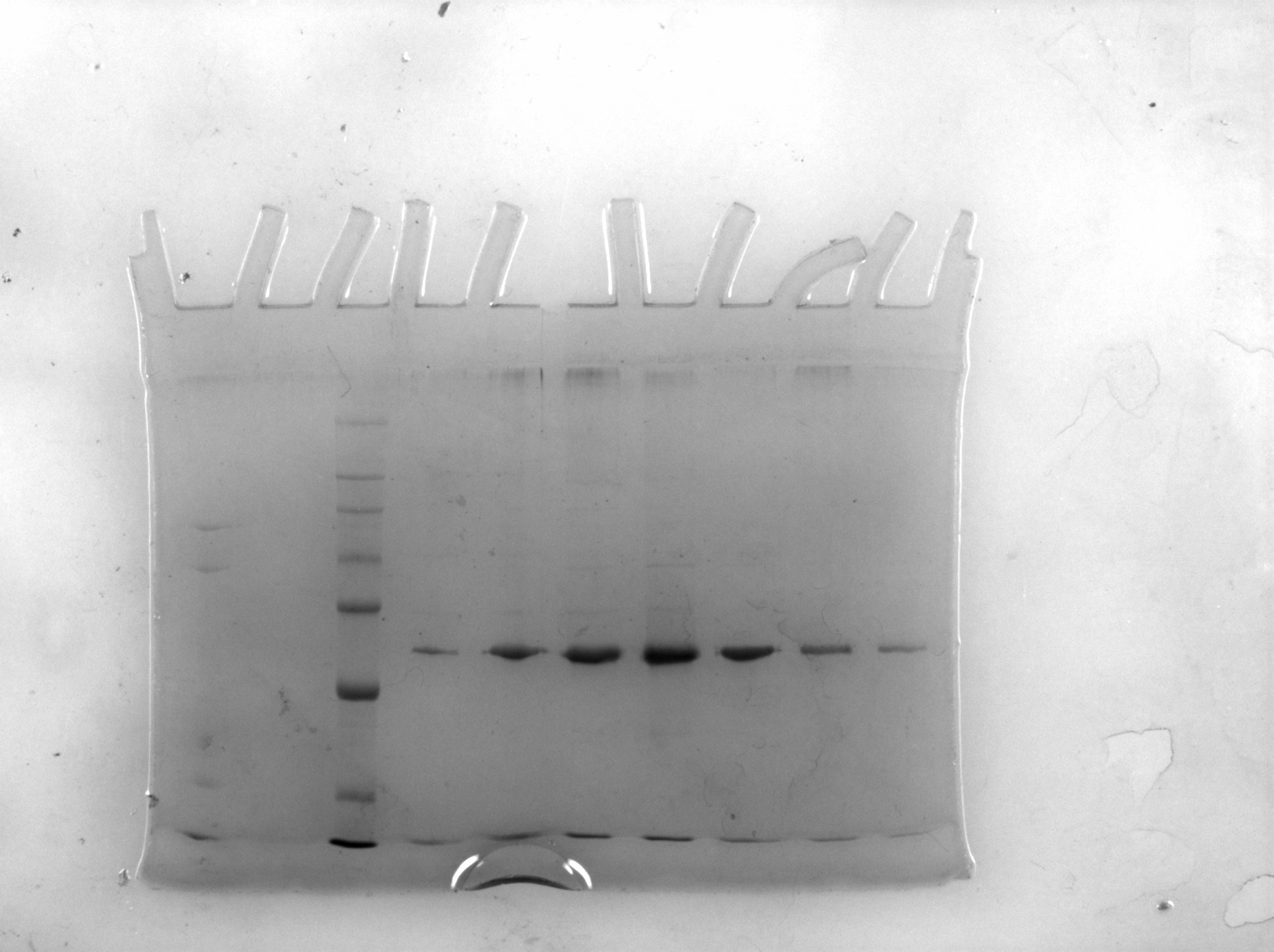

Supplement: Figure S9 — The eluted Cbs2 protein was purified by anion-exchange chromatography. Peak Cbs2 38.66 mS/cm represents the Cbs2 protein that was eluted when the conductance was 38.66 mS/cm, The peak Cbs2 protein was estimated by SDS-PAGE, The SDS-PAGE results showed that the Cbs2 protein was further purified, as shown in Fig. 2D. [file peerj-09-10901-s009.jpg]

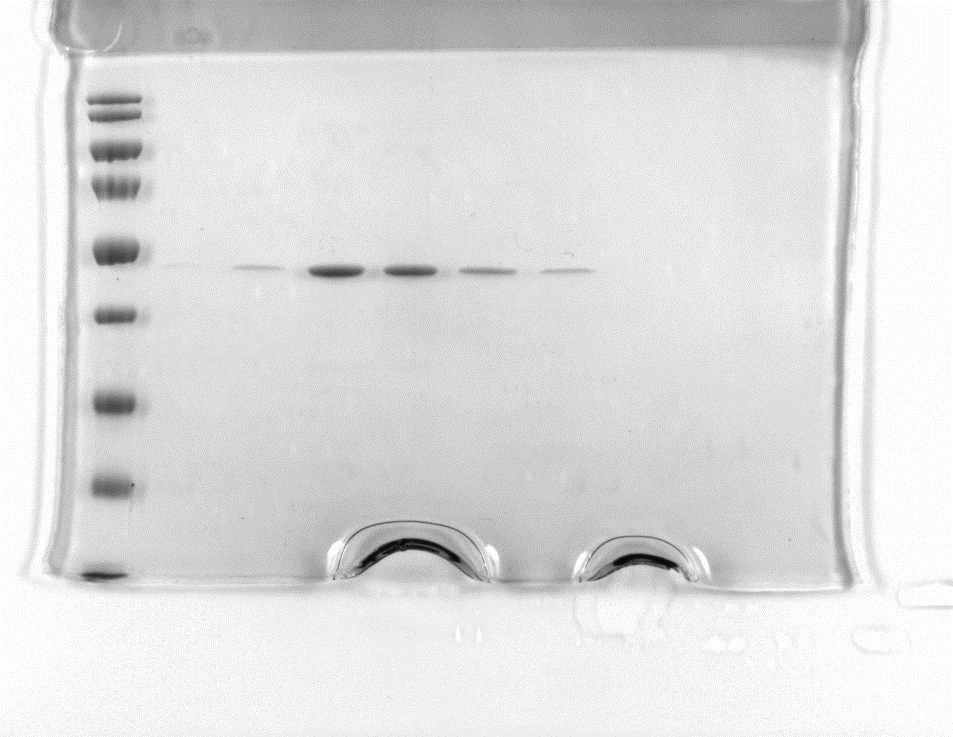

Supplement: Figure S10 — The collected peak protein was further purified using a gel filtration chromatography column. The peak Cbs2 protein was estimated by SDS-PAGE, The SDS-PAGE results showed that the protein was pure and concentrated, as shown in Fig. 3B. [file peerj-09-10901-s010.png]

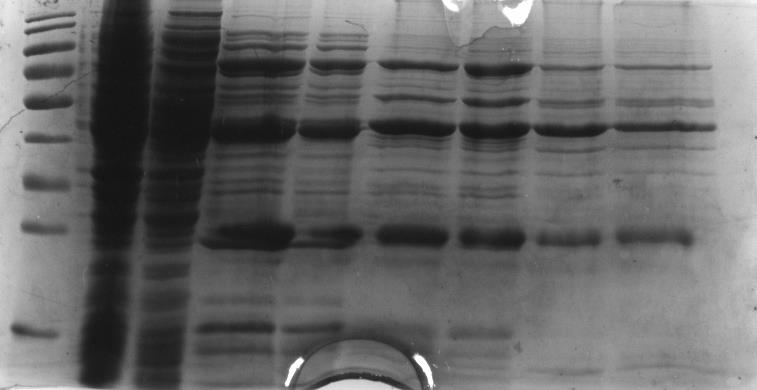

Supplement: Figure S11 — The SDS-PAGE results showed that some impurities were unable to be washed away even after using a washing buffer that contained up to 70 mM imidazole, as shown in Fig. S2A. [file peerj-09-10901-s011.jpg]

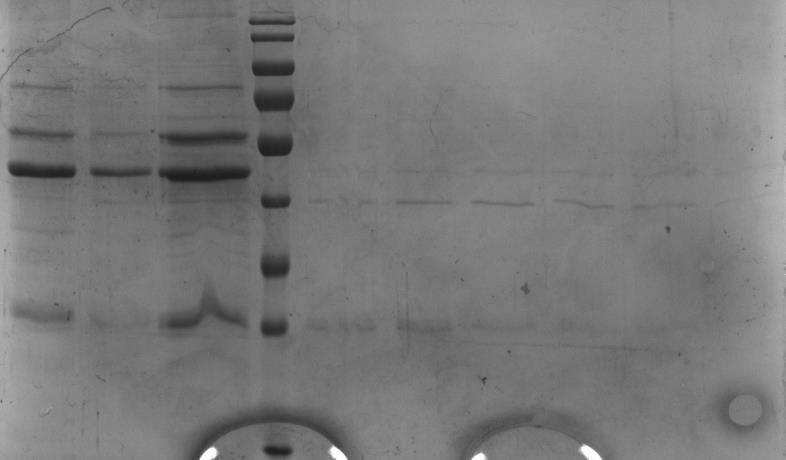

Supplement: Figure S12 — The SDS-PAGE results showed that there were still some impurities in the eluted Cbs2 protein, as shown in Fig. S2B. [file peerj-09-10901-s012.jpg]

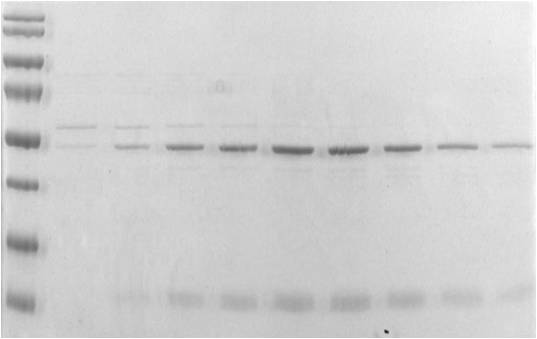

Supplement: Figure S13 — The SDS-PAGE results showed that the purity of the protein after ion exchange was still not high, and the protein behavior was not good, as shown in Fig. S2D. [file peerj-09-10901-s013.jpg]
