# Supplementary material for: Expression, purification, crystallization and preliminary X-ray crystallographic studies of a mitochondrial membrane-associated protein Cbs2 from Saccharomyces cerevisiae
Source: PeerJ. 2021 Feb 17;9:e10901. doi: 10.7717/peerj.10901 (PMC7896505; doi:10.7717/peerj.10901)
Supplement: Table S1 — The cloning details were shown, including primers, vector, Cbs2 gene sequences and competent cells. [file peerj-09-10901-s014.docx]

Table S1

| Information | Cbs2 cloning |
| --- | --- |
| Source organism | *Saccharomyces cerevisiae* (strain ATCC 204508) |
| DNA source | CAA31876 |
| Forward primer | 5’ GAAATTCATATGTCAAGCTCAATACCTAG3’ |
| Reverse primer | 5’ GAAATTCTCGAGTCACAGGTAATGATAATCTAG3’ |
| Cloning vector | p28 (a modification of PET-28a, with a hexahistidine tag MGHHHHHH at the N-terminus) |
| Plasmid host | *E. coli* DH5α competent cell |
| Expression host | *E. coli* C43 competent cell |
| Complete amino acid sequence | MGHHHHHHMGSMSSSIPRVYSLGNSAMTYLLALRIAQLPSQPKVPSVVLLLNDQKKLNRFLNNDSKIIVKSSNNNKETYHRQFMASCVPPILSNGEIAPIENLIVSDPSSKFITAQLSKYNKSLRPETNILFLNPSLNLLEHLHRYRWRFDEARPNLFMGFTTPVDVGTIHQEFQLSLKVKGRIQFHIAKIDGFPRMSSTGKSASLSLRGDRQKNEKENNAFYKLFREISRLRSGIGSDLVSFDLHVHGFQDLFFTELEKLILESCTEPLLAVYDCVYKKELLKIPGAQDIIKKLISEQLSIIDRSYPSLNTNPNYSVIFDKERIFSLVMRDLEVNGHKRAKLAQSLNQLNQTNINELNGFFVSLGKYKKC NCKWNDILLTLIKGKQFITKQKALDYHYL |
