# Supplementary material for: Expression, purification, crystallization and preliminary X-ray crystallographic studies of a mitochondrial membrane-associated protein Cbs2 from Saccharomyces cerevisiae
Source: PeerJ. 2021 Feb 17;9:e10901. doi: 10.7717/peerj.10901 (PMC7896505; doi:10.7717/peerj.10901)
Supplement: Table S2 — The protein yield (mg/L of cell culture) and purity (%) of each purification step. [file peerj-09-10901-s015.docx]

Table S2

| Purification step | protein yield (mg/L of cell culture) | purity (%) |
| --- | --- | --- |
| Total solubilized proteins | 112 | - |
| After purification by Ni-NTA  chromatography | 3.21 | 73 |
| After Hitrap Q anion-exchange chromatography | 1.37 | 91 |
| After Superdex 200 10/300 gel-filtration chromatography | 0.86 | 99 |
