# Supplementary material for: Expression, purification, crystallization and preliminary X-ray crystallographic studies of a mitochondrial membrane-associated protein Cbs2 from Saccharomyces cerevisiae
Source: PeerJ. 2021 Feb 17;9:e10901. doi: 10.7717/peerj.10901 (PMC7896505; doi:10.7717/peerj.10901)
Supplement: Table S3 — The crystallization details are shown, including method, plate type, temperature, protein concentration, crystallization kits, composition of reservoir solution, volume and ratio of drop and volume of reservoir. [file peerj-09-10901-s016.docx]

Table S3

| Information | Cbs2 Crystallization |
| --- | --- |
| Method | Vapour diffusion |
| Plate type | sitting-drop |
| Temperature (K) | 289K |
| Protein concentration | 10 mg/mL |
| Buffer composition of protein | 20 mM Tris-HCl, pH 7.5, 100 mM NaCl and 0.05 mM DDM |
| crystallization kits | Crystal Screen, Crystal Screen 2, ProPlex, Salt RX, Index, PEG/Ion , PEG/Ion 2 |
| Composition of reservoir solution | 0.2 M Potassium phosphate dibasic and 18% Polyethylene glycol 3350 |
| Volume and ratio of drop | 2.4 μL, 1:1 |
| Volume of reservoir | 200 μL |
